# Old and New Glycopeptide Antibiotics: Action and Resistance

**DOI:** 10.3390/antibiotics3040572

**Published:** 2014-11-04

**Authors:** Elisa Binda, Flavia Marinelli, Giorgia Letizia Marcone

**Affiliations:** 1 Department of Biotechnology and Life Sciences, University of Insubria, Varese 20100, Italy; E-Mails: flavia.marinelli@uninsubria.it (F.M.); giorgia.marcone@uninsubria.it (G.L.M.); 2 The Protein Factory, Interuniversity Centre Politecnico di Milano, ICRM CNR Milano and University of Insubria, Milan 21100, Italy

**Keywords:** glycopeptides, resistance, *van* genes, *Nonomuraea* sp. ATCC 39727, dalbavancin

## Abstract

Glycopeptides are considered antibiotics of last resort for the treatment of life-threatening infections caused by relevant Gram-positive human pathogens, such as *Staphylococcus aureus*, *Enterococcus* spp. and *Clostridium difficile*. The emergence of glycopeptide-resistant clinical isolates, first among enterococci and then in staphylococci, has prompted research for second generation glycopeptides and a flurry of activity aimed at understanding resistance mechanisms and their evolution. Glycopeptides are glycosylated non-ribosomal peptides produced by a diverse group of soil actinomycetes. They target Gram-positive bacteria by binding to the acyl-d-alanyl-d-alanine (d-Ala-d-Ala) terminus of the growing peptidoglycan on the outer surface of the cytoplasmatic membrane. Glycopeptide-resistant organisms avoid such a fate by replacing the d-Ala-d-Ala terminus with d-alanyl-d-lactate (d-Ala-d-Lac) or d-alanyl-d-serine (d-Ala-d-Ser), thus markedly reducing antibiotic affinity for the cellular target. Resistance has manifested itself in enterococci and staphylococci largely through the expression of genes (named *van*) encoding proteins that reprogram cell wall biosynthesis and, thus, evade the action of the antibiotic. These resistance mechanisms were most likely co-opted from the glycopeptide producing actinomycetes, which use them to avoid suicide during antibiotic production, rather than being orchestrated by pathogen bacteria upon continued treatment. *van*-like gene clusters, similar to those described in enterococci, were in fact identified in many glycopeptide-producing actinomycetes, such as *Actinoplanes teichomyceticus*, which produces teicoplanin, and *Streptomyces toyocaensis*, which produces the A47934 glycopeptide. In this paper, we describe the natural and semi-synthetic glycopeptide antibiotics currently used as last resort drugs for Gram-positive infections and compare the *van* gene-based strategies of glycopeptide resistance among the pathogens and the producing actinomycetes. Particular attention is given to the strategy of immunity recently described in *Nonomuraea* sp. ATCC 39727. *Nonomuraea* sp. ATCC 39727 is the producer of A40926, which is the natural precursor of the second generation semi-synthetic glycopeptide dalbavancin, very recently approved for acute bacterial skin and skin structure infections. A thorough understanding of glycopeptide immunity in this producing microorganism may be particularly relevant to predict and eventually control the evolution of resistance that might arise following introduction of dalbavancin and other second generation glycopeptides into clinics.

## 1. Natural Glycopeptide Antibiotics

Glycopeptide antibiotics (GPAs) are frequently used to treat life-threatening infections caused by multidrug-resistant Gram-positive pathogens, such as *Staphylococcus aureus*, *Enterococcus* spp. and *Clostridium difficile*. They are drugs of last resort against methicillin-resistant *Staphylococcus aureus* (MRSA), which is nowadays a major cause of community-acquired infections and results in high morbidity and mortality rates in hospital-acquired infections [1]. First-generation GPAs are natural products composed of glycosylated non-ribosomal heptapeptides produced by a diverse group of actinomycetes [2,3].

**Figure 1:**
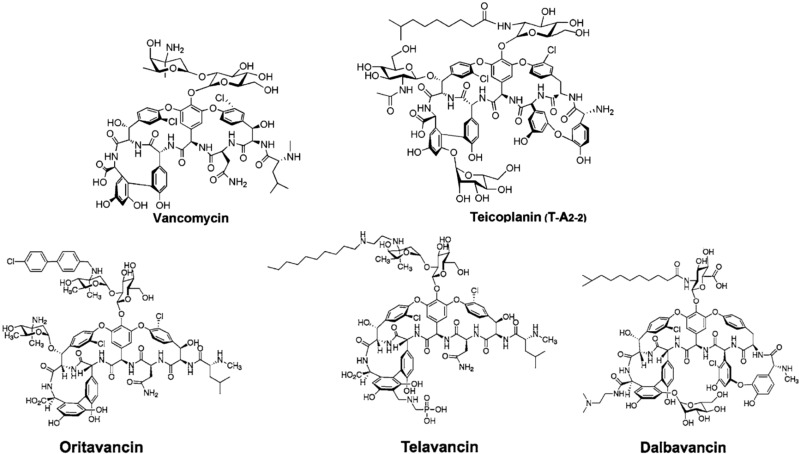
Structures of natural and semi-synthetic glycopeptide antibiotics (GPAs). Vancomycin and teicoplanin are natural products. In the case of teicoplanin, the clinically used antibiotic is a mixture of five lipoglycopeptide molecules differing in the length (C10-C11) and branching of the fatty acid tail, whose main component, the one reported in the figure, bears the 8-methylnonanoic (*iso*-C10:0) acid and is named T-A_2-2_. Oritavancin and telavancin are semi-synthetic second generation GPAs belonging to the vancomycin family. Dalbavancin is the semi-synthetic derivative of the teicoplanin-like A40926.

The common structural motif is a core heptapeptide scaffold containing aromatic amino acids that have undergone extensive oxidative cross-linking and decoration with different moieties, such as sugar residues, chlorine atoms and lipid chains. Vancomycin and teicoplanin (Figure 1) represent the first generation of clinically important GPAs. Vancomycin, produced by the actinomycete *Amycolatopsis orientalis*, was first introduced in clinics in 1958, whereas teicoplanin, produced by *Actinoplanes teichomyceticus*, was first reported in 1978 and then introduced in clinical use in Europe in 1988 and in Japan in 1998 [1,4]. These two main antimicrobial GPA scaffolds (Figure 1) contain proteinogenic (Tyr, Leu, Asn, Ala and Glu) and non-proteinogenic amino acids (4-hydroxyphenylglycine, 3,5-dihydroxyphenylglycine and β-hydroxytyrosine). Five of the seven residues in vancomycin are aromatic, and two are aliphatic, while all seven are aromatic in teicoplanin [5]. Consequently, the number of oxidative cross-links between aromatic amino acids are three in vancomycin and four in teicoplanin, conferring the peculiar structural conformation representing the binding pocket for the cellular antibiotic target [6].

Many more GPAs with amino acid sequences that differ from teicoplanin and vancomycin exist in nature and continue to be discovered by natural product screening (for an extensive review, see [2,7]). The diverse heptapeptide scaffolds undergo further biosynthetic modifications that include glycosylation, halogenation, methylation and, in some cases, sulfation and sugar acylation. Tailoring of GPAs can alter their chemical and biological properties by increasing solubility, imparting stability, affecting dimerization, determining membrane attachment, limiting conformational flexibility, avoiding degradation and evading resistance [7,8,9]. New GPA scaffolds and related tailoring enzymes have been recently found by screening environmental DNA libraries by metagenomics [10,11,12]. 

Vancomycin and teicoplanin (Figure 1) contain two chlorine atoms and are glycosylated by a disaccharide on amino acid residue 4 in vancomycin or by three monosaccharides at residues 4, 6 and 7 in teicoplanin. The glucosamine moiety attached to residue 4 in teicoplanin is acylated by a fatty acid chain. Clinically used teicoplanin is a mixture of five lipoglycopeptide molecules differing in the length (C10-C11) and branching of the fatty acid tail, whose main component is the one bearing the 8-methylnonanoic (iso-C10:0) acid and named T-A_2-2_ [13,14].

GPAs inhibit bacterial cell wall synthesis by binding to the d-Ala-d-Ala dipeptide terminus of the peptidoglycan (PG) precursors, sequestering the substrate from transpeptidation and transglycosylation reactions in the late extracellular stages of PG cross-linking. The d-Ala-d-Ala complex with GPAs is stabilized by an array of hydrophobic van der Waals contacts and five hydrogen bonds lining the antibiotic binding pocket [6,15]. Cross-linked PG is needed for the appropriate tensile strength of the cell wall. Thus, GPAs action ultimately results in destabilizing the cell wall integrity, with bacterial cell death occurring presumably due to osmotic insult. The requirement for direct access of GPAs to the target PG precursor explains the selective inhibition of Gram-positive bacteria. Gram-positive bacteria expose PG precursors on the external surface of the cytoplasmic membrane, whereas Gram-negative bacteria are protected by the presence of an outer lipopolysaccharide membrane impermeable to large biomolecules [3].

The antibacterial spectrum of teicoplanin activity against Gram-positive bacteria is similar to that of vancomycin, but teicoplanin shows an increased potency, particularly against some clinical isolates belonging to *Staphylococcus*, *Streptococcus* and *Enterococcus* genera [16,17]. Acylation of teicoplanin confers a lipophilic nature to the antibiotic that is suggested to impart superior antimicrobial activity and pharmacokinetics in comparison to vancomycin [3,18,19,20]. Consequently, most of the second generation semi-synthetic GPAs were prepared introducing hydrophobic moieties in the heptapeptide scaffold in order to confer increased membrane anchoring ability, leading to improved drugs [6,20,21].

## 2. Semi-Synthetic Glycopeptide Antibiotics

The spread of resistance to vancomycin in enterococci since 1988 and the emergence of high-level GPA resistance in clinical isolates of MRSA since 2002 have prompted the search for second generation drugs belonging to the GPA class. Second generation GPAs are semi-synthetic derivatives of natural products. 

Telavancin (Vibativ) (Figure 1, Table 1), firstly approved by the Food and Drug Administration (FDA) for clinical use in 2009, is a derivative of vancomycin and differs from the parent compound by the addition of a hydrophobic and a hydrophilic group to the vancomycin structure. The length of the hydrophobic side chain was chosen to reach a compromise between optimized activity against MRSA (8–10 C) and vancomycin resistant enterococci (VRE) (12–16 C). The hydrophilic properties of the phosphonate group improve the adsorption, distribution, metabolism and excretion profile of the compound. Pharmacological studies suggest that the enhanced activity of telavancin *versus* vancomycin on *Streptococcus pneumoniae*, *Staphylococcus aureus* (to a lesser extent) and enterococci (including VRE) results from a complex mechanism of action, which involves perturbation of lipid synthesis and, possibly, membrane disruption [16,21,22]. 

**Table 1:** Second generation GPAs approved and/or in development [23].

Drug	GPA Precursor	Microbiological Spectrum	Main Clinical Indication	Status
Oritavancin (Orbactiv)	Chloroeremomycin	MRSA ^1^, VRSA ^1^, VRE ^1^	ABSSSI ^2^	approved by FDA ^3^ in 2014
Telavancin (Vibativ)	Vancomycin	MRSA ^1^, MSSA ^1^, VSE ^1^, *Streptococcus pyogenes*	cSSSI ^2^	approved by FDA ^3^ in 2009
		*Staphylococcus aureus*	HABP/VABP ^2^	approved by FDA ^3^ in 2013
Dalbavancin (Dalvance)	A40926	MRSA ^1^, MSSA ^1^, *Streptococcus pyogenes*	ABSSSI ^2^	approved by FDA ^3^ in 2014

^1^ MRSA, methicillin-resistant *Staphylococcus aureus*; VRSA, vancomycin-resistant *Staphylococcus aureus*; VRE, vancomycin-resistant enterococci; MSSA, methicillin-susceptible *Staphylococcus aureus*; VSE, vancomycin-susceptible enterococci; ^2^ ABSSSI, acute bacterial skin and skin structure infections; cSSSI, complicated skin and skin structure infections; HABP/VABP, hospital-acquired and ventilator-associated bacterial pneumonia; ^3^ FDA, Food and Drug Administration.

Oritavancin (Orbactiv) (Figure 1, Table 1) is the *N*-alkyl-*p*-chlorophenyl-benzyl derivative of chloroeremomycin produced by the actinomycete *Amycolatopsis orientalis*. Chloroeremomycin differs from vancomycin by the glycosylation pattern on amino acid residues 4 and 6. Although oritavancin presents a general spectrum of activity comparable to that of vancomycin, it offers considerable advantages in terms of intrinsic activity (especially against streptococci) and remains insensitive to the resistance mechanisms developed by staphylococci and enterococci; it is also active against *Clostridium difficile* [24]. The FDA recently accepted oritavancin for clinical use (Table 1). According to recent investigations on the mode of action, the biaryl group is involved in causing cell membrane depolarization. The superior activity against Gram-positive pathogens, including those resistant to vancomycin, is due to this dual-action mechanism, either inhibiting cell wall biosynthesis or affecting membrane integrity [25]. 

Dalbavancin (Figure 1, Table 1) is a semi-synthetic derivative of the teicoplanin-like molecule A40926, which was isolated from the actinomycete *Nonomuraea* sp. ATCC 39727 collected from an Indian soil in the mid-1980s [26]. In comparison with teicoplanin, A40926 lacks the saccharide moiety on the amino acid residue 6. It also differs from teicoplanin due to the presence of an acylaminoglucuronic acid on amino acid 4 instead of the acylglucosamine. Other structural differences between A40926 and teicoplanin include the terminal methylamino group, the position of one chlorine atom and the length of the fatty acid chain [27]. A40926 was used as a scaffold for an extensive program of chemical derivatization producing several clinical leads [27]; dalbavancin is the dimethylaminopropyl amide derivative (Figure 1) [5,28]. Dalbavancin shows an increased *in vitro* activity, compared to vancomycin, towards most Gram-positive pathogen bacteria, as well as an extremely long half-life, permitting once-weekly intravenous dosing [21,29]. On May 23 2014, clinical used dalbavancin (Dalvance) was approved by the FDA (Table 1).

The success of these three second generation GPAs as drug candidates and their potentiated antibacterial activities in comparison to vancomycin are stimulating further efforts in studying the mechanism of action/resistance and developing novel derivatives [20,30]. Due to the progresses achieved by total synthesis of GPAs by the group of Boger since 1999, single atom changes in vancomycin are today possible and pave the way to rationally redesign GPAs exhibiting potent antibacterial activity against VRE and MRSA [6]. The novel tailoring enzymes discovered by the group of Brady in environmental libraries offer a successful strategy for generating libraries of GPA variants [10,11,12]. 

## 3. The *van* Gene Clusters in Pathogens

Resistance to GPAs has manifested itself largely in enterococci through the expression of genes (named *van*) encoding proteins that reprogram cell wall biosynthesis and, thus, evade the action of the antibiotics. The onset of vancomycin resistance was long-delayed in comparison to all other antibiotics. The first case of vancomycin resistance was reported in *Enterococcus faecium* [31], but since that time, VRE have become increasingly widespread throughout the world and are nowadays found as multi-resistant opportunistic pathogens in hospitals and also in the environment (food animals) [1,32]. The detailed mechanism of *van* gene-mediated GPA resistance in enterococci was elucidated by Courvalin, Walsh and their co-workers in the 1990s (for an extensive review, see [3,33]). In the two most prominent manifestations of resistance (VanA and VanB phenotypes, Table 2), the PG precursor is remodeled to the terminal d-Ala-d-Lac, incorporating an ester linkage in place of the amide of d-Ala-d-Ala. The replacement of a dipeptide with a depsipeptide removes one of the hydrogen bonding interactions and leads to lone pair-lone pair repulsion, reducing by 1000-fold the affinity of GPAs to their molecular target and resulting in a corresponding 1000-fold loss in antimicrobial activity [34,35]. 

**Figure 2:**
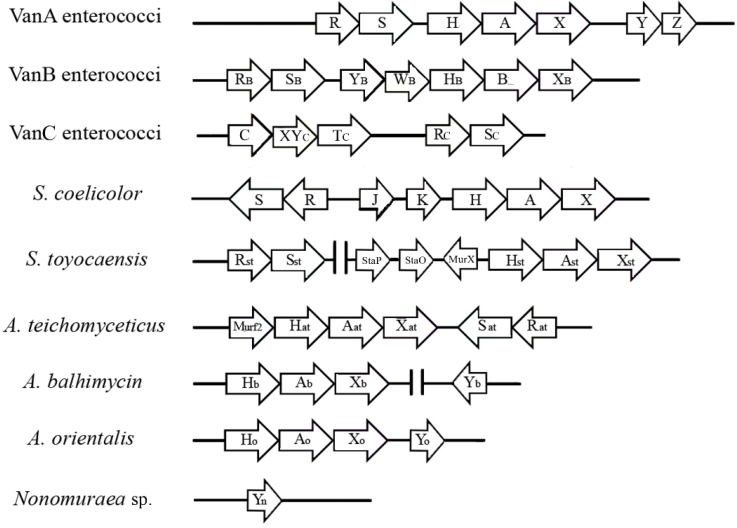
Comparison of resistance gene organization in enterococci (VanA, VanB and VanC phenotypes) in *Streptomyces coelicolor* and in GPAs-producers. Two parallel vertical lines indicate that genes that are not present in contiguous regions (figure modified from Yim *et al.* [2]).

VanA-type resistance is characterized by high-level inducible resistance to both vancomycin and teicoplanin (Table 2) and is mediated by transposable elements, such as Tn*1546* [36]. Tn*1546* carries three genes encoding essential enzymes VanH, VanA, and VanX that remodel the PG precursor from d-Ala-d-Ala to d-Ala-d-Lac (Figure 2). VanH is a dehydrogenase that converts pyruvate into d-lactate [37]; VanA is a d-Ala-d-Lac ligase [34]; and VanX is a d-Ala-d-Ala dipeptidase that cleaves any residual d-Ala-d-Ala dipeptide [38,39], ensuring that PG precursors terminate mostly in d-Ala-d-Lac. Tn*1546* also encodes two accessory proteins, VanY and VanZ, that are not required for, but contribute to, high-level resistance to vancomycin and teicoplanin, respectively [36]. VanZ confers low-level teicoplanin resistance in the absence of the other resistance proteins by an unknown mechanism [40]. VanY is a d,d-carboxypeptidase that hydrolyses the d-Ala C-terminal residue of PG precursors synthesized by using the d-Ala-d-Ala dipeptides that have escaped VanX hydrolysis [36,41].

The *vanA* operon expression is regulated by two genes, *vanR* and *vanS*, located upstream from *vanH* (Figure 2), that form a two-component signaling cascade [42,43,44,45]. Initiated by a ligand-induced dimerization of a cell surface His-kinase (VanS), it activates the signaling transcription factor VanR by phosphorylation and dimerization. In turn, the activated VanR binds to DNA and induces the expression of *van* genes [46]. The species responsible for inducing VanS dimerization and activation has been the subject of intense study: direct binding of the GPAs to VanS or its activation by cell wall intermediates that accumulate as a result of antibiotic action is still debated [44,47,48]. Despite the *vanA* and *vanB* operons having similar genetic organization and *ca.* 60% sequence identity in VanHAX proteins, the sequences of the sensor kinases and response regulators of VanA and VanB-type strains are only 23% and 34% identical at the amino acid level. The sensor kinase of VanB-type enterococci, named VanS_B_, responds to different signals in comparison to VanS [49], being activated by vancomycin, but not by teicoplanin. In fact, vancomycin and teicoplanin induce resistance in VanA enterococci, whereas VanB strains sense vancomycin, but are resistant to teicoplanin (Table 2).

GPA resistance in enterococci can also results from substitution of the terminal d-Ala-d-Ala with d-Ala-d-Ser. The substitution leads only to a moderate (six-fold) decrease in the affinity of vancomycin for its target and accordingly to low-level resistance to the antibiotic [50]. Isolates of *E. gallinarum*, *E. casseliflavus* and *E. flavescens* (VanC phenotype) are intrinsically resistant to low levels of vancomycin, due to their constitutive production of PG precursors ending in d-Ala-d-Ser, but they remain sensitive to teicoplanin (Table 2). Three enzymes are required for the synthesis of d-Ala-d-Ser ending PG precursors: a racemase (VanT) that converts l-Ser to d-Ser, a ligase (VanC) that synthesizes d-Ala-d-Ser and a bi-functional d,d-dipeptidase/d,d-carboxypeptidase (VanXY_C_) that removes the d-Ala-d-Ala synthesized by the host ligase and removes the C-terminal d-Ala residue from the natural PG precursors [51]. Differently from VanX (d-Ala-d-Ala peptidase) and VanY (d,d-carboxypeptidase), VanXY_C_ hydrolyzes both di- and penta-peptide. The genes encoding a two-component sensor (VanS_C_) and regulatory (VanR_C_) system are located downstream from the operon encoding VanC, VanXY_C_ and VanT_C_. The basis of the constitutive phenotype is due to mutations in the sensor VanS_c_ [51,52,53].

More recently, other types of *van* operon structures (*vanD*, *vanE*, v*anG*, *vanL*, *vanM*, *vanN*) and corresponding resistance phenotypes have been reported in enterococci (Table 2) [33,54,55,56]. They are designated according to the characteristics of the key ligase that encodes either a d-Ala-d-Lac or a d-Ala-d-Ser ligase [36]. In addition to *vanA* and *vanB*, the d-Ala-d-Lac ligase group includes *vanD* and *vanM* genes. The d-Ala-d-Ser ligase group includes *vanC*, *vanE*, v*anG*, *vanL* and *vanN*. The d-Ala-d-Lac-type operons are located on either plasmids or the chromosome; the d-Ala-d-Ser-type operons have been generally detected in the chromosome, except for the case of *vanN*, which was found in a plasmid in *E. faecium* [57]. Variable GPA-resistance phenotypes are associated with the presence of different operons, whose expression is inducible (*vanA*, *vanB*, v*anG*, *vanE*, *vanL*, *vanM*) or constitutive (*vanC*, *vanD*, *vanN*) (Table 2).

**Table 2:** Features of GPA resistance in enterococci. PG, peptidoglycan.

Microorganisms	GPA Resistance Phenotype	Level of Resistance	MIC (mg/L) of GPAs	Location of *van* Genes	Transcription of *van* Genes	C-terminal of Modified PG Target	References
*E. faecalis* *E. faecium*	VanA	High	Vancomycin 64–100 Teicoplanin 16–512	Plasmid Chromosome	Inducible	d-Ala-d-Lac	[44,45,46]
*E. faecalis* *E. faecium*	VanB	Variable	Vancomycin 4–1000 Teicoplanin 0.5–1	Plasmid Chromosome	Inducible	d-Ala-d-Lac	[33,36,44]
*E. gallinarum* *E. casseliflavus* *E. flavescens*	VanC	Intrinsic resistance, low level	Vancomycin 2–32 Teicoplanin 0.5–1	Chromosome	Constitutive	d-Ala-d-Ser	[33,49,54]
*E. faecalis* *E. faecium*	VanD	Moderate	Vancomycin 64–128 Teicoplanin 4–64	Chromosome	Constitutive	d-Ala-d-Lac	[33,53]
*E. faecalis*	VanE	Low	Vancomycin 8–32 Teicoplanin 0.5	Chromosome	Inducible	d-Ala-d-Ser	[33]
*E. faecalis* *E. faecium*	VanG	Low	Vancomycin 16 Teicoplanin 0.5	Chromosome	Inducible	d-Ala-d-Ser	[33]
*E. faecalis*	VanL	Low	Vancomycin 8 Teicoplanin susceptible	Chromosome	Inducible	d-Ala-d-Ser	[54]
*E. faecium*	VanM	Variable	Vancomycin > 256 Teicoplanin 0.75	Plasmid Chromosome	Inducible	d-Ala-d-Lac	[56]
*E. faecium*	VanN	Low	Vancomycin 16 Teicoplanin 0.5	Plasmid	Constitutive	d-Ala-d-Ser	[55,57]

The first case of *van* gene-mediated resistance in high vancomycin-resistant *S. aureus* (VRSA) was detected in 2002. The VanA phenotype-resistant strain was from a dialysis patient in Michigan co-infected by a vancomycin-resistant *E. faecalis*, implicating horizontal gene transfer mediated by Tn*1546* [58,59]. This event was alarming, since *S. aureus* is responsible for severe infections and toxinoses in both the hospital environment and the community, and for almost three decades, vancomycin has been increasingly used to treat *S. aureus* infections, due to the global emergence of MRSA, which is resistant to multiple drug classes [1]. Fortunately, only a few additional VRSA isolates have been reported thus far, including 13 isolates from the United States, 16 from India, 3 from Iran and 1 from Pakistan [60,61], and in no case VRSAs were involved in severe bacteremic infections. Competition growth experiments, in the absence of the inducing vancomycin between MRSA recipient and isogenic VRSA transconjugant, revealed a disadvantage for the transconjugant, accounting, in part, for the low level of dissemination of VRSA clinical isolates [62]. In addition to that, it has been indicated that the restriction modification system of *S. aureus* limits the transfer of resistance genes between isolates of different *S. aureus* lineages [63].

## 4. The *van* Gene Clusters in the Producing Actinomycetes

GPAs are produced by filamentous actinomycetes, which are soil-dwelling mycelial high G-C content Gram-positive bacteria [64]. Their complex cell life cycle consists of vegetative growth followed by the formation of aerial hyphae and, ultimately, spore formation, the last allowing both dispersal and persistence under unfavorable conditions. The onset of morphological differentiation generally coincides with the production of two-thirds of the commercially-available antibiotics, including GPAs [5,65]. Antibiotic-producing actinomycetes possess mechanisms to avoid suicide by their own toxic products. The extensive review by Cundliffe and Demain [66] covered the different strategies adopted, ranging from target-based ones (*i.e.*, modification of normal drug receptors or *de novo* synthesis of the latter in the drug-resistant form) to those based on the adoption of molecular shielding and/or efflux that prevent drug-target interactions.

The first evidence that *van* gene-mediated resistance occurred in the producing actinomycetes dated back to the late 1990s, when orthologues of enterococcal *vanHAX* were cloned from two GPAs-producing actinomycetes: *Streptomyces toyocaensis* NRRL15009, which is the producer of the sulfonated sugar-free teicoplanin-like GPA, known as A47934, and the vancomycin producer *Amycolatopsis orientalis* C329.2 [67]. The predicted amino acid sequences were found to be highly similar to those found in VRE: 54% to 61% identity for VanH, 59% to 63% for VanA and 61 to 64% identity for VanX [67,68]. The d-Ala-d-Ala ligase from *S. toyocaensis* showed d-Ala-d-Lac ligase activity in cell-free extracts of *S. lividans* transformed with the *vanA*-like gene and confirmed the predicted enzymatic activity [67]. Similar gene sequences were then identified in other GPAs-producers, such as *A. orientalis* (chloroeremomycin producer), *A. orientalis* subsp. *lurida* (ristocetin producer), *Amycolatopsis coloradensis* subsp. *labeda* (teicoplanin and avoparcin producer), *A. balhimycina* (balhimycin producer) and *A. teichomyceticus* (teicoplanin producer), suggesting that actinomycetes may represent the original source of the *van* genes involved in the synthesis of resistant PG precursors in pathogens [68,69,70,71].

Sequencing of the A47934 biosynthetic gene cluster (*sta*) of *S. toyocaensis* revealed the 5' orthologues of the *vanHAX* cluster organized as a presumptive operon (*vanH_st_A_st_X_st_*), indicating that resistance genes are coupled to the biosynthetic ones in this GPA-producing organism [72]. The co-regulation of resistance and biosynthetic genes involved in A47934 production was confirmed by insertional inactivation of *vanA_st_*: the resulting mutant was more susceptible to the GPA, and its production was delayed by 16 hours, until the cells entered into the stationary phase of growth and were no longer sensitive to the GPA action [72]. Associated with *vanH_st_A_st_X_st_*, the biosynthetic gene cluster *sta* includes three putative accessory genes (Figure 2): *murX_st_*, encoding a predicted d-Ala-d-Ala adding enzyme; *staO*, encoding a protein with high homology to the FemABX family responsible for the production of pentaglycine inter-muramyl chain peptide in the PG [73]; and *staP*, a putative membrane protein with unclear function (see the paragraph below on *Streptomyces coelicolor). vanR_st_* and *vanS_st_* are not in close proximity to the *vanH_st_A_st_X_st_* genes as in VRE and VRSA, but they are separated by approximately 20 kb, outside the *sta* biosynthetic cluster [72]. The sensor protein VanS_st_ is quite divergent from enterococcal VanS and VanS_B_ (15% overall identity in pairwise comparisons) [48]. Interestingly, *S. toyocaensis* is resistant to A47934, but it is sensitive to both vancomycin and teicoplanin [74] (Table 3), and it was more recently demonstrated that vancomycin does not trigger VanS_st_ autophosphorylation and does not induce GPA resistance in *S. toyocaensis* [47]. 

Studies based on the sequencing of fosmid clones or whole genomes have allowed the annotation of *van*-like genes in other GPAs-producing actinomycetes. Sequencing of the biosynthetic gene cluster for teicoplanin (*tcp* or *tei*) of *A. teichomyceticus* [69,70] revealed the presence at one end of a set of contiguous genes putatively involved in GPA resistance: *murF2*, the *vanHAX* homologues and two genes codifying for a sensory kinase and a response regulator of a putative two-component signal transduction system (Figure 2). Cloning of these genes in *S. coelicolor* increased the host GPA resistance, albeit at a reduced level [75]. Notwithstanding the lack of genetic tools for manipulating *A. teichomyceticus*, Beltrametti *et al.* [76] demonstrated by quantitative PCR and liquid chromatography-mass spectrometry (LC-MS) of PG precursors that *vanH_at_A_at_X_at_* are organized in an operon, whose expression is constitutive and determines the exclusive production of PG precursors ending in d-Ala-d-Lac depsipeptides. Taking advantage of the high degree of identity (77%) between the VanS sequences of *S. coelicolor* (see paragraph below) and *A. teichomyceticus*, two significant amino acid substitutions were identified in the ATPase domain of VanS_at_, which were considered to be crucial for the VanS phosphorylase activity in *S. coelicolor*; since their mutation resulted in constitutive *van* gene expression and subsequent constitutive resistance [77]. As reported in VRE (see the paragraph above), the constitutive resistance phenotype is associated with specific mutations in VanS [52,53]. In *A. teichomyceticus*, a mutated VanS homolog conferring constitutive GPA resistance may have been selected as an adaptation to teicoplanin production [76]. Consistently, *A. teichomyceticus* is constitutively resistant to GPAs, with MICs of teicoplanin and vancomycin of 25 and 90 µg/mL, respectively (Table 3).

**Table 3:** Features of GPA-resistance in actinomycetes.

Microorganisms	Produced GPA	MIC (mg/L) of GPAs	Location of *vanHAX* Genes	Transcription of *vanHAX* Genes	C-terminal ^1^ of PG Target in Absence of Inducer	C-terminal ^1^ of PG Target in Presence of Inducer	References
*Streptomyces coelicolor*	none	Vancomycin >100 Teicoplanin <0.5	Chromosome	Inducible by vancomycin	d-Ala-d-Ala	d-Ala-d-Lac	[47,78,79,80]
*Streptomyces toyocaensis*	A47934	Vancomycin <0.25 Teicoplanin <0.25 A47934 >5	Chromosome, A47934 Cluster	Inducible by A47934	d-Ala-d-Ala	d-Ala-d-Lac	[47,68,72,74]
*Actinoplanes teichomyceticus*	Teicoplanin	Vancomycin 90 Teicoplanin 25	Chromosome, *tei* Cluster	Constitutive	d-Ala-d-Lac	d-Ala-d-Lac	[69,70,75,76]
*Amycolatopsis balhimycin*	Balhimycin	Vancomycin n.r.^2^ Teicoplanin n.r.^2^ Balhimycin >100	Chromosome, out of *bal* Cluster	Constitutive	d-Ala-d-Lac	d-Ala-d-Lac	[71]
*Nonomuraea* sp. ATCC 39727	A40926	Vancomycin 30 Teicoplanin 0.9 A40926 4	n.d. ^3^	Inducible by A40926	d-Ala ^4^	d-Ala ^4^	[81,82]

^1^ In all of the strains (except *Nonomuraea* sp. ATCC 39727), the reported C-terminus indicates the terminal dipeptide or the depsipeptide in the pentapeptide stem of the PG precursor; ^2^ n.r., not reported. ^3^ n.d., not detectable: *vanHAX* genes have not been found in *Nonomuraea* sp. ATCC 39727 [80]. ^4^ In *Nonomuraea* sp. ATCC 39727, the predominant PG precursor is the tetrapeptide UDP-MurNAc-l-Ala-d-Glu-meso-Dap-d-Ala.

In *A. balhimycin*, which synthesizes the GPA balhimycin (which differs only in its glycosylation pattern from vancomycin), *vanHAX* gene homologues were found 2 Mb away from the biosynthetic gene cluster (*bal*) [71]. These genes are expressed constitutively, resulting in a constitutive production of PG precursors terminating in d-Ala-d-Lac, as demonstrated by quantitative PCR and LC-MS of PG precursors (Table 3). Within the *bal* cluster, homologues of known enterococcal resistance genes *vanS*, *vanR* and *vanY* were initially annotated and named *vnlR*, *vnlS* and *vanY_b_*. Further studies on the phenotypes of mutated strains showed that the VnlR/S two-component system is not involved in GPA resistance: RT-PCR analyses confirmed similar expression levels of *vanH*_b_*A*_b_*X*_b_ and *vanY_b_* in the wild-type and in a *vnlR* deletion mutant [71]. VanY_b_ is a d,d-carboxypeptidase, which increases the level of GPA resistance as described in enterococci harboring the *vanHAX* genes, but in contrast to the enterococcal enzyme, VanY_b_ lacks a predicted membrane association site [71]. Heterologous expression of VanY_b_ in *S. coelicolor* increased the level of balhimycin resistance in the host (50 μg/mL *versus* 10 μg/mL in an agar diffusion test). In contrast, the expression of VanY_b_ in the *ΔvanR/S* mutant of *S. coelicolor* [83], which is susceptible to vancomycin (since the *vanHAX* genes cannot be induced due to the absence of the two-component regulatory system; see the paragraph below), did not confer balhimycin resistance in this background. These data indicate that *vanY_b_* is an accessory gene that requires functional VanHAX proteins to increase the level of resistance [71].

More recently, the sequence assembly s from metagenomic samples [10,11,12] has allowed the identification of the following GPA clusters: VEG (vancomycin-like environmental DNA-derived gene cluster), TEG (teicoplanin-like environmental DNA-derived gene cluster), CA37, CA878, CA915 and two teicoplanin-type molecules with eSNaPD (environmental Surveyor of Natural Product Diversity) identification numbers 15 and 26 [10,11,12]. In these environmental clusters, the position of genes that encode each class of enzyme required for the biosynthesis of a GPA is conserved: interestingly, resistance genes (*vanHAX*-like) are grouped together at the beginning of the cluster, followed by nonribosomal peptide synthetase genes [11]. 

## 5. The Model System Streptomyces Coelicolor

*S. coelicolor* is genetically the model species of antibiotic-producing filamentous actinomycetes [84]. It harbors a *vanHAX* gene cluster, but it does not produce any GPA [78,84]. *S. coelicolor* lives in soil and shares the same ecological niche with GPA-producing actinomycetes; thus, it seems likely that it might have acquired *van* genes, gaining a selective advantage [77]. The genetic cluster consists of seven genes (*vanRSJKHAX*) divided into four transcription units (*vanRS*, *vanJ*, *vanK* and *vanHAX*) that confer high inducible resistance to vancomycin, but not to teicoplanin (the VanB enterococcal phenotype) (Figure 2, Table 3). The predicted products of the *S. coelicolor vanH*, *vanA* and *vanX* genes are close homologues (ranging from 60% to 65%) of their counterparts from VRE [78] and, when switched on, reprogram PG biosynthesis replacing d-Ala-d-Ala terminating PG precursors in d-Ala-d-Lac-ending ones [83]. 

VanS and VanR proteins of *S. coelicolor* are indeed quite divergent from their enterococcal equivalents [78]. VanS of *S. coelicolor* shows a high homology (65%–77% overall identity in pairwise comparisons) with equivalent sensor proteins in *S. toyocaensis* and in *A. teichomyceticus* [47], and its function has been intensively studied [47,77,80]. The *ΔvanR/S* mutant of *S. coelicolor* is susceptible to vancomycin, since this GPA cannot induce *vanHAX* genes, due to the absence of the two-component regulatory system [77,78]. Studying a set of *S. coelicolor* mutants, the following model of the two-component regulation of GPA resistance was proposed: on exposure to vancomycin, VanS’s activity switches from being a phosphatase to a kinase, and the resulting accumulation of phospho-VanR activates transcription from *van* promoters and induces vancomycin resistance. In the absence of drug, however, the constitutive phosphorylation of the response regulator VanR mediated by small-molecule phosphor-donor acetyl phosphate is suppressed by the phosphatase activity of VanS [77]. Koteva *et al.* [47] showed that a vancomycin phoaffinity probe binds to *S. coelicolor* VanS and that binding is required for the expression of the *van* genes and for resistance to vancomycin, suggesting that for at least some VanS sensor kinases associated with the VanB phenotype, the antibiotic itself is the ligand that induces drug resistance. More recently, Kwun *et al.* [80] undertook a series of *in vivo* experiments, which indicate that *S. coelicolor* VanS is activated by vancomycin in complex with the d-Ala-d-Ala termini of PG precursors. This may represent a new intermediate model between the alternative ones previously proposed, *i.e.*, the direct binding of the antibiotic to the sensor domain or the indirect induction by intermediate cell wall metabolites accumulating as a result of antibiotic action. The two accessory genes, *vanJ* and *vanK*, were not found in VRE [78,79]. VanJ is a membrane protein that increases resistance to teicoplanin in *S. coelicolor*, but is not required for resistance to vancomycin [79]. Interestingly, VanJ is orthologous to *S. toyocaensis* StaP (75% amino acid sequence identity), previously identified in the A47934 biosynthetic cluster. A thorough comprehension of VanJ function in the cell wall metabolism needs further elucidation, which might be relevant to obtaining a more complete understanding of the mode of action of teicoplanin (Table 2) [81]. 

The *vanK* gene is indeed essential for vancomycin resistance: it is a member of the Fem proteins family, which add the glycine/s to the stem pentapeptide of PG precursors [78]. In *S. coelicolor*, the branch is a single glycine residue, and in the absence of vancomycin, this residue is added by the FemX enzyme. However, the constitutive FemX activity in *S. coelicolor* can recognize only PG precursors terminating in d-Ala-d-Ala as a substrate [83]. VanK is required for vancomycin resistance, because it is the only enzyme that can add glycine to PG precursors terminating in d-Ala-d-Lac. Production of precursors lacking a side-chain would be lethal, because this would prevent cross-linking of the PG by transpeptidase, leading to cell lysis [83]. VanK enzyme is important for GPA resistance, not just in *S. coelicolor*, since an orthologous (*staO*) was previously identified in the A47934 biosynthetic cluster [72]. In contrast, the absence of orthologues of *vanK* in vancomycin-resistant clusters of pathogens implies that their FemX can recognize precursors terminating in d-Ala-d-Ala or d-Ala-d-Lac.

## 6. The Case of *Nonomuraea* sp. ATCC 39727

The *Nonomuraea* sp. ATCC 39727 produces the teicoplanin-like A40926 that is the precursor of the semi-synthetic dalbavancin [26,28]. Homologues of *vanHAX* genes were not found by sequencing the A40926 biosynthetic gene cluster (*dbv*) [85]. They were not also identified by mining *Nonomuraea* genome by southern hybridization using degenerate primers and v*anH_at_A_at_X_at_* genes from *A. teichomyceticus* as probes [81]. Moreover, preliminary data from genome sequencing of *Nonomuraea* sp. ATCC 39727 indicated a lack of significant homology with *van* genes from *A. teichomyceticus* and enterococci [81]. 

*Nonomuraea* sp. ATCC 39727 shows a moderate resistance to GPAs (Table 3); interestingly, A40926 MIC is 4 μg/mL during vegetative growth, but this increases to *ca.* 20 μg/mL during A40926 production, suggesting the induction of an alternative mechanism of self-protection. Thus far, the sole resistant determinant identified in *Nonomuraea* sp. is the *vanY*-like gene (*vanY*_n_) found in the *dbv* cluster and encoding a zinc-dependent d,d-carboxypeptidase capable of removing the terminal d-Ala residue of pentapeptide in the PG precursors, leaving tetrapeptide that is not recognized by GPAs [81]. Accordingly, analysis of PG precursors by LC-MS revealed the predominant presence of the tetrapeptide UDP-MurNAc-l-Ala-d-Glu-*meso*-Dap-d-Ala and only traces of the corresponding pentapeptide terminating in d-Ala-d-Ala [79]. Consistently, a *vanY*_n_ null mutant of *Nonomuraea* sp. demonstrated a reduced level of GPA resistance and accumulated the pentapeptide PG precursors, and its complementation restored resistance and the cell wall phenotype [81,82]. Recently, Hugonnet *et al.* [86] reported that the PG of *Nonomuraea* sp. ATCC 39727 was partially cross-linked by an l,d-transpeptidase (LTD) that uses the tetrapeptide acyl donors supplied by the VanY_n_ action on the pentapeptide PG precursors. In many bacteria, especially among *Actinomycetales*, LTDs catalyze the formation of 3→3 linkages, complementing the action of the better known d,d-transpeptidases of the penicillin binding protein (PBP) family that use precursors containing a pentapeptide stem and catalyze the formation of 4→3 cross-links connecting the fourth amino acid residue of the acyl donor to the third position of the acyl acceptor. In *Nonomuraea* sp. ATCC 39727 during exponential growth, 49% of the cross-links were generated by l,d-transpeptidation, whereas in the stationary phase, the proportion of 3→3 cross-links was lower (31%) [86].

Recombinant VanY_n_ was heterologously expressed in and purified from either *S. venezuelae* or *Escherichia coli* [87,88]. It is a membrane-bound bi-functional d,d-peptidase and d,d-carboxypeptidase, containing conserved motifs (SxHxxGxAxD and ExxH) involved in the coordination of zinc and in the active site that are typical of VanY and VanX zinc-dependent d,d-carboxypeptidases and d,d-peptidases characterized in GPA-resistant enterococci [38,39,41,89]. The dual function of VanY_n_ most closely resembles the one of VanXY_C_ from VanC-type *Enterococcus gallinarum,* which combines the specificity of VanX and VanY as it hydrolyzes both the d-Ala-d-Ala and the PG precursor analogs ending in d-Ala [90]. Surprisingly, increasing concentrations of penicillin G and ampicillin inhibited VanY_n_ activity, whereas zinc-dependent VanX peptidases and VanY carboxypeptidases from enterococci are resistant to β-lactams [39,89]. In enterococci, both zinc-dependent d,d-carboxypeptidases and low molecular weight penicillin binding proteins (LMW PBPs) catalyze the same reaction, but have a completely different protein domain architecture [91]. Similar to classical VanY enzymes, VanY_n_ lacks the canonical Ser-x-x-Lys motif found in the active sites of PBPs; thus, how β-lactams inhibit VanY_n_ activity is worthy of further investigations. Despite the high identity of the conserved sequences belonging to the active sites, the overall identity between VanY_n_ and the previously described VanY and VanX enzymes in enterococci is moderate, ranging from 25% to 46% [87]. VanY_n_ shows 48% sequence identity with VanY_b_ d,d-carboxypeptidase identified in the *bal* cluster of *A. balhimycina* [71], but it differs from VanY_b_ in the mass and cellular localization [82,87]. In the recent work by Meziane-Cherif *et al.* [92], the phylogenetic reconstruction of the metallo peptidase M15 family showed that VanY_n_ is close to, but does not branch within, the group of VanY (including VanY_b_) and VanXY from enterococci (subfamily M15B). This suggests that VanY_n_ can represent an ancestral enzyme with a role in self-resistance, then recruited for resistance in other bacteria through structural changes that affect its substrate specificity and activity. Other authors taking advantage of the tools developed to genetically manipulate *Nonomuraea* sp. [93,94], recently varying *vanY_n_* gene dosage and expressing *vanH_at_A_at_X_at_* from the teicoplanin producer *A. teichomyceticus* in *Nonomuraea* sp. Knocking out, complementing a *vanY_n_* mutant or duplicating *vanY_n_* influenced antibiotic resistance, confirming the role of this gene in conferring moderate resistance to GPAs in *Nonomuraea* sp. due to the incomplete conversion of the PG pentapeptide precursors to the tetrapeptides. Heterologous expression of *vanH_at_A_at_X_at_* increased A40926 resistance in *Nonomuraea* sp. and resulted in the marked accumulation of the UDP-MurNAc-pentadepsipeptide (terminating in d-Lac) [82].

## 7. Conclusions

The core *vanHAX* genes have been detected in all of the published GPAs-producers (except the A40926 producer). The last announcement comes from the genome sequencing of *A. orientalis* that produces vancomycin: in this case, *vanHAX* is located in the front of the vancomycin biosynthetic genes [95]. In addition, *vanHAX* have been recently found in GPA-like gene clusters identified in environmental DNA by metagenomics tools [2,10,11,12]. In most of the reported cases (except the balhimycin producer), *vanHAX* is part of, or very close to, the GPAs-biosynthetic cluster, suggesting a coordinated regulation of GPA production and resistance. As reported in the case of *A. balhimycina* and *A. teichomyceticus*, these producers bypass the VanRS regulation, and the *vanHAX* genes are constitutively expressed. This might be a safety mechanism to protect nonproducing cells living in a population with neighboring GPA producer cells, during the critical transition from the growth phase towards antibiotic production. Interestingly, in a recent work based on the resistance-guided isolation of novel GPA-producing actinomycetes, Thaker *et al.* identified two distinct *Streptomyces* sp. strains (WAC1420 and WAC4229) that produce a novel GPA, named pekiskomycin [96]. Their draft genomes show identical biosynthetic cluster organization, except for recombinations in WAC4229, resulting in the loss of *vanRS*. Thus, it appears that among GPA producers, the lack of *vanRS*-based regulation may be a quite common feature. In contrast, stringent regulation of resistance gene expression represents a more common trait of not-producing bacteria, such as *S. coelicolor*, and resistant pathogens that remodel their cell wall biosynthesis only if exposed to GPAs. The ubiquitous presence of regulatory systems in these bacteria indicates that there is a selective advantage for the production of alternate types of precursors in response to environmental conditions. 

In enterococci, staphylococci and actinomycetes, *vanHAX* are always present in the same order and are co-transcribed, and the VanHAX proteins show high levels of amino acid sequence identity across different resistant genotypes. Regulatory genes may be placed upstream or downstream of the *vanHAX* genes in enterococci or far away in the genome of actinomycetes. The lack of sequence similarity in the sensor domains of VanS kinases reflects different modes of recognition of GPAs. Most gene clusters include accessory resistance genes that are specific for each cluster and whose function is generally less known. *vanY* are considered ancillary genes in enterococci, but apparently, they are relevant resistance determinants in some GPA-producing actinomycetes. In enterococci, they are much more diverse than VanHAX, and their genes occupy different positions in different resistant genotypes, *i.e.*, upstream or downstream of *vanHAX*. Some GPA-producing actinomycetes possess, alternatively, *vanHAX* or *vanY* genes, and some others combine *vanHAX* and *vanY*. Since, in VRE, levels of resistance to GPAs are determined by the extent of elimination of PG precursors ending in d-Ala-d-Ala, *vanHAX* and *vanY* combination perhaps emerged and was selected in actinomycetes and pathogens as the most protective solution, whereby VanX and VanY enzymes act in sequence to eliminate PG precursors ending with d-Ala-d-Ala.

Concluding, several lines of evidence indicate that *van* gene clusters have been generated by the recruitment of different genes or sets of genes from the GPA-producing actinomycetes, including the variations in the guanosine plus cytosine content in different portions of the cluster, the variable percentage of sequence identity between the encoded proteins, the variation in the order of genes and the contribution of unrelated accessory proteins. Studying resistance determinants in the actinomycetes may provide new insights into their evolution and may also contribute to an early warning system for emerging resistance mechanisms due to the exposure to old and new GPAs. This may also provide new targets to design specific inhibitors to be used in combination with GPAs.
